# Neuromyelitis Optica Spectrum Disorder Presenting With Organizing Pneumonia: A Case Report and Literature Review

**DOI:** 10.7759/cureus.84602

**Published:** 2025-05-22

**Authors:** Yosuke Takeuchi, Teruaki Masuda, Mami Otsuka, Atsunobu Takeda, Noriyuki Kimura

**Affiliations:** 1 Department of Neurology, Faculty of Medicine, Oita University, Yufu, JPN; 2 Department of Ophthalmology, Oita University, Yufu, JPN

**Keywords:** aquaporin-4, area postrema syndrome, extra-central nervous system symptoms, neuromyelitis optica spectrum disorder, organizing pneumonia

## Abstract

Neuromyelitis optica spectrum disorder (NMOSD) is an inflammatory disease of the central nervous system (CNS) that can result in severe neurological sequelae. Aquaporin-4, a cause of complement-mediated inflammation in NMOSD, is expressed on alveolar epithelial cells and in the CNS, and organizing pneumonia (OP) is recognized as an extra-CNS symptom in NMOSD. We herein present a case of NMOSD following OP. The patient initially presented with area postrema syndrome, and the coexistence of OP made the diagnosis of NMOSD challenging. A review of previously reported cases of NMOSD with OP suggests that careful attention to area postrema syndrome as an onset symptom is needed for the early diagnosis and treatment of NMOSD in male and older patients with OP.

## Introduction

Neuromyelitis optica (NMO) is an inflammatory disease involving the central nervous system (CNS), and anti-aquaporin-4 (AQP4) antibodies are specific for the diagnosis of NMO [1]. The term "NMO spectrum disorder (NMOSD)" has been advocated as a broader concept for AQP4 antibody-seropositive diseases, and consensus diagnostic criteria of NMOSD with anti-AQP4 antibody need at least one core clinical characteristic, including optic neuritis, acute myelitis, area postrema syndrome (APS), acute brainstem syndrome, symptomatic narcolepsy, acute diencephalic clinical syndrome, and symptomatic cerebral syndrome [2]. APS is a distinctive syndrome involving intractable nausea, vomiting, or hiccups due to a medullary lesion. In patients with NMOSD, activities of daily living are severely impaired owing to CNS involvement, and early diagnosis and treatment are required to prevent neurological sequelae.

AQP4 is also expressed in organs other than the CNS, including the lung epithelium [3]. Additionally, some cases of organizing pneumonia (OP), which is one of the diffuse interstitial lung diseases with intra-alveolar buds of connective tissue, have been reported as an extra-CNS symptom of NMOSD [4-11]. However, the coexistence of NMOSD and OP is rare, and the clinical features of NMOSD with OP have not been clarified.

We herein report a case of NMOSD following OP and review its clinical features in NMOSD with OP to aid in early recognition and diagnosis.

## Case presentation

**Figure 1:**
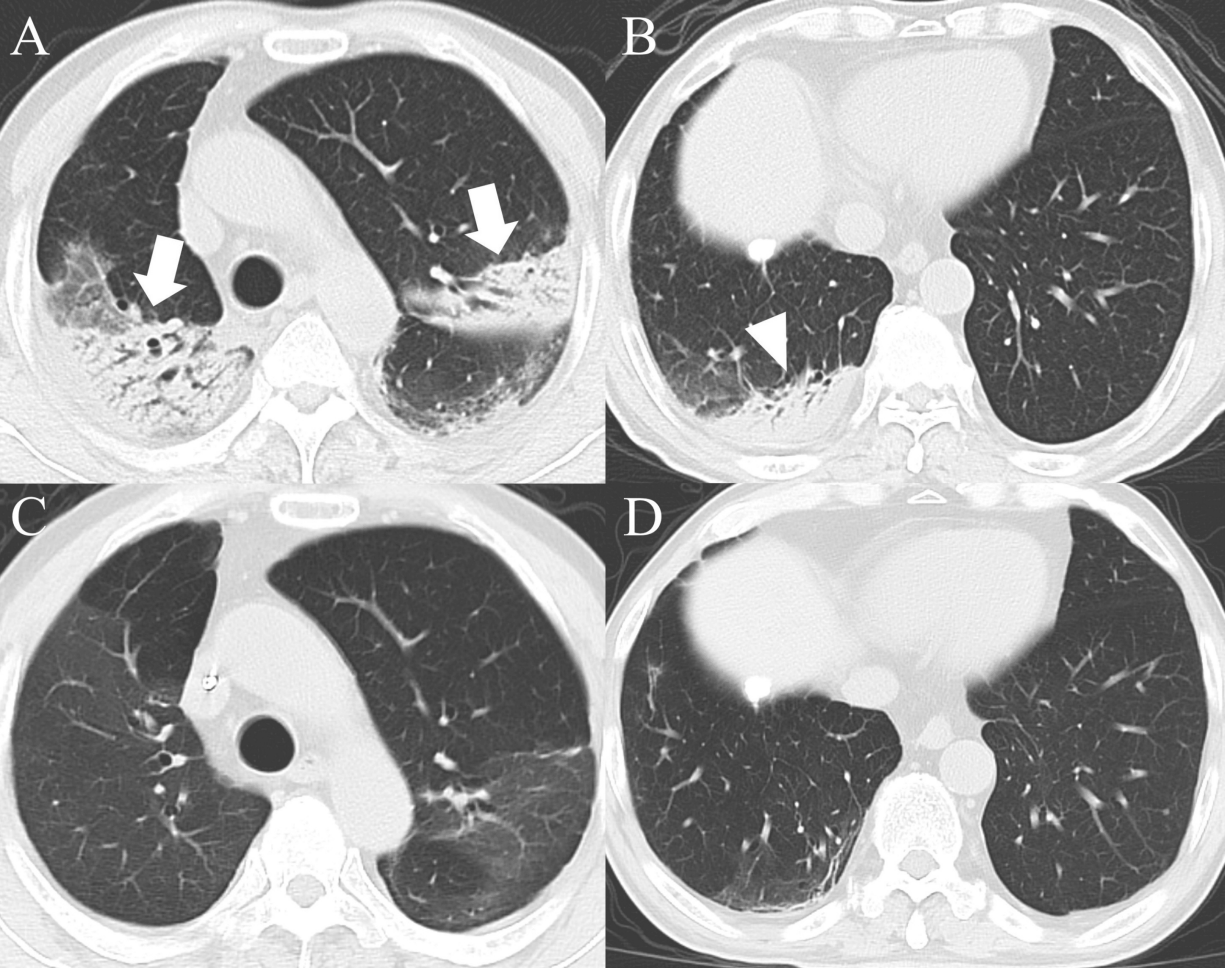
Chest computed tomography. The chest CT showed multiple bilateral consolidations during the first admission (A, arrows; B, arrowhead). No recurrence of the lung lesions was observed on the second admission (C, D).

A 72-year-old man with nausea was admitted to a local hospital. He started presenting with continuous coughing eight days earlier, and chest computed tomography (CT) demonstrated multiple bilateral consolidations on admission (Figures 1A, 1B).

**Figure 2:**
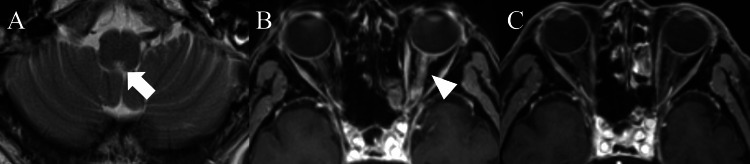
Brain magnetic resonance imaging. The brain MRI showed a slightly hyperintense lesion in the area postrema on day 25 (A, arrow). Contrast-enhanced brain MRI demonstrated enhancement of the left optic nerve on day 64 (B, arrowhead), which improved after immunotherapy (C).

On day 14, he began experiencing frequent hiccups. The lung lesions showed no response to antibacterial agents and improved after steroid therapy (methylprednisolone 40 mg/day on day 23, followed by oral prednisolone 20 mg/day). Brain magnetic resonance imaging (MRI) on day 25 showed a slightly hyperintense lesion in the area postrema (Figure 2A).

Although bronchoscopy was not performed because the patient did not provide consent, he was diagnosed with OP, and prednisolone was tapered to 10 mg/day. The nausea and hiccups gradually resolved.

**Figure 3:**
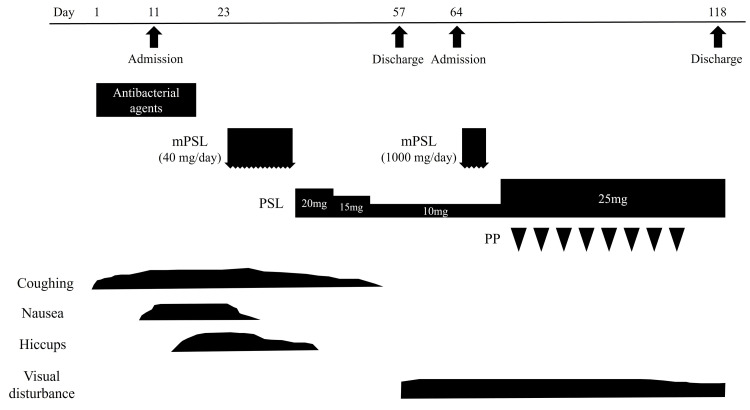
Clinical course of the present case. Nausea and hiccups occurred following organizing pneumonia, and both improved after the administration of steroid therapy. However, during the tapering of prednisolone, the patient experienced visual loss in the left eye, leading to the diagnosis of neuromyelitis optica spectrum disorder. The addition of intensive immunotherapy led to a partial improvement in the patient's visual acuity. mPSL: methylprednisolone, PSL: prednisolone, PP: plasmapheresis

However, he experienced left visual disturbance on day 57, which progressed to blindness two days later, and was admitted to the department of ophthalmology in our hospital. Neurological investigation revealed a loss of the left pupillary light reflex, paresthesia in bilateral lower extremities, urinary urgency, and constipation. No weakness, pyramidal signs, or cerebellar ataxia were observed. A contrast-enhanced brain MRI demonstrated enhancement of the left optic nerve on day 64 (Figure 2B). No abnormal intensities were seen in the spinal cord. Blood examination revealed anti-AQP4 antibody positivity by enzyme-linked immunosorbent assay, and he was diagnosed with NMOSD based on the international consensus diagnostic criteria [2]. Anti-myelin oligodendrocyte glycoprotein antibodies, anti-nuclear antibodies, anti-neutrophil cytoplasmic antibodies, anti-SS-A/Ro antibodies, and anti-SS-B/La antibodies tested negative. Chest CT showed no recurrence of OP (Figures 1C, 1D). Because steroid pulse therapy (methylprednisolone 1000 mg/day, five days) had no effect, the patient was transferred to the department of neurology, and plasmapheresis was added. While enhancement of the left optic nerve improved (Figure 2C), his left visual acuity was hand motion at the time of discharge. The clinical course is shown in Figure 3.

## Discussion

Herein, we described a case of NMOSD following OP. The patient presented with nausea and protracted hiccups as initial symptoms of NMOSD, which had been misdiagnosed as being caused by frequent coughing due to OP, possibly delaying the diagnosis of NMOSD. As a result, the delayed introduction of plasmapheresis might cause irreversible damage to the optic nerve.

AQP4, one cause of complement-mediated inflammation in NMOSD, is diffusely expressed on alveolar epithelial cells as well as astrocytes in the CNS, and the deposition of membrane attack complex, probably caused by the AQP4-IgG binding in the alveolar epithelial cells surrounding OP lesions, has been reported [3,4,10]. These findings suggest that the comorbidity of NMOSD and OP is not a mere coincidence, and OP is one of the extra-CNS symptoms in NMOSD. However, as in our case, the coexistence of OP can make the diagnosis of NMOSD challenging, and an investigation of the clinical features of NMOSD with OP is needed for early diagnosis.

We searched PubMed and Google Scholar with a combination of the keywords "neuromyelitis optica" and "organizing pneumonia" and "aquaporin-4" for studies published until December 31, 2024. We reviewed the articles, which included individual clinical data, and identified 14 cases of NMOSD with OP [4-11]. The clinical findings of reported cases of NMOSD with OP and the present case are shown in Table 1.

**Table 1:** Literature review of 15 cases of neuromyelitis optica spectrum disorder with organizing pneumonia. *: area postrema syndrome NMOSD: neuromyelitis optica spectrum disorder, OP: organizing pneumonia, M: male, F: female, C: cervical cord, Th: thoracic cord, PP: plasmapheresis, ND: not described

Case number	Authors, year, reference	Age/sex	Initial neurological symptoms	Optic neuritis	Myelitis	Periods between the onset of NMOSD and OP	Acute treatments	Outcome of neurological deficits
1	Nieva, et al. [4]	75/M	Abdomen paresthesia, gait disturbance	Absent	Present (C4 to Th10)	6 months, OP → NMOSD	High-dose corticosteroid	Partially effective
									2	Nieva, et al. [4]	74/F	Sensory disturbance in the lower extremities	Absent	Present (C2 to Th1, Th7 to Th9)	2 years, OP → NMOSD	High-dose corticosteroid	Effective
3	Asato, et al. [5]	76/M	Hiccups*	Absent	Present (C1-2, C4-6)	Simultaneous	High-dose corticosteroid, PP	Effective									
									4	Oliveira, et al. [6]	73/F	Right hemiparesis	Present (right)	Present (C1 to C7)	Simultaneous	High-dose corticosteroid	Effective
5	Rimpa, et al. [7]	77/F	Numbness, paresthesia, and weakness of both lower limbs and the left upper limb	Absent	Present (C1 to C2, C4 to C7, Th6 to Th9)	2 months, OP → NMOSD	High-dose corticosteroid	Effective									
									6	Ra, et al. [8]	75/F	Bilateral lancinating pain in the trunk with allodynia	Present (bilateral)	Present (Th3 to Th8)	2 weeks, OP → NMOSD	High-dose corticosteroid	Effective
7	Shafiq, et al. [9]	14/F	Visual loss of the right eye	Present (right)	Absent	Simultaneous	High-dose corticosteroid, PP	Poor									
									8	Furube, et al. [10]	80s/M	ND	ND	ND	1 month	High-dose corticosteroid	ND
9	Furube, et al. [10]	40s/M	ND	ND	ND	Simultaneous	High-dose corticosteroid	ND
10	Furube, et al. [10]	70s/M	ND	ND	ND	Simultaneous	High-dose corticosteroid	ND
11	Furube, et al. [10]	40s/F	ND	ND	ND	Simultaneous	High-dose corticosteroid	ND
12	Lai, et al. [11]	75/F	Nausea*, hiccups*	Absent	Present (C1 to Th2)	17 days, OP → NMO	High-dose corticosteroid, PP	Effective
									13	Lai, et al. [11]	46/F	Nausea*, hiccups*	Absent	Absent	Simultaneous	High-dose corticosteroid	Effective
14	Lai, et al. [11]	68/F	Nausea*, numbness, and burning pain over the torso, paresthesia in both feet	Absent	Present	Simultaneous	High-dose cyclophosphamide	ND
15	New case, 2025	72/M	Nausea*	Present (left)	Absent	8 days, OP → NMO	High-dose corticosteroid, PP	Partially effective

Six (40.0%) patients were male, and 11 (73.3%) were classified as late-onset NMOSD; both were more frequent than the overall prevalence of NMOSD (11.6-30.3% and 20-28%, respectively) [12]. The initial neurological symptoms were mainly myelopathy and APS in 54.5% and 45.5% of patients, respectively. APS has been reported to occur in approximately 10% of patients with NMOSD at the onset [13]. Our review demonstrated a higher frequency of APS as an onset manifestation, which may be a characteristic of NMOSD with OP. APS is often misdiagnosed as a symptom of other systemic diseases, and the recognition of APS as a manifestation of NMOSD is important [14]. APS is likely to be a key sign in the early diagnosis of NMOSD with OP.

In contrast, visual disturbance was a rare initial symptom in only one (9.1%) case of NMOSD with OP. Although optic neuritis is a core manifestation that occurs in 52.8% of patients with NMOSD, late-onset cases display less optic neuritis compared to early-onset cases [13,15]. The onset age of NMOSD patients in our review was older, which may explain the low frequency of visual symptoms in patients with NMOSD who have OP. However, four (36.4%) patients presented with optic neuritis during the disease, and it is important not to miss the appropriate time for the treatment of optic neuritis.

Eight (53.3%) patients developed NMOSD and OP simultaneously; in other cases, NMOSD occurred following OP. The median periods between NMOSD and OP onset were 0 days (from 0 days to two years). The onset of both diseases is often close in time, and the neurological manifestations of NMOSD may be masked by systemic symptoms of OP, leading to a delay in the diagnosis of NMOSD. We should pay attention to neurological manifestations, including APS and myelopathy, to detect NMOSD earlier in male or older patients with OP.

This case report has certain limitations. The small sample size and reliance on case reports might introduce selection bias. Publication bias due to underreporting of mild cases should also be considered. These biases may have affected the characteristics of NMOSD with OP, which contrast with general demographics in NMOSD, including onset age, male prevalence, and the frequency of APS as an initial symptom. Therefore, a prospective study with a large sample size is needed to address these issues.

## Conclusions

This case report highlighted the clinical features of NMOSD with OP. AQP4 is expressed in alveolar epithelial cells as well as the CNS, suggesting a potential relationship between NMOSD and OP. NMOSD is more likely to occur simultaneously with or following OP in male, older-onset patients. Careful attention to neurological manifestations, especially APS, and the investigation of anti-AQP4 antibodies are needed for the early diagnosis and treatment of NMOSD in patients with OP.
